# Advancing anthracnose resistance in dry beans through the transition from traditional to computational breeding efforts

**DOI:** 10.3389/fpls.2026.1809294

**Published:** 2026-05-15

**Authors:** Aashvi Patel, Sajal Ahlawat, Philip Lorenc, Maryam Vazin, Masoud Maleki, Mohsen Yoosefzadeh Najafabadi

**Affiliations:** 1 Department of Plant Agriculture, University of Guelph, Guelph, ON, Canada; 2 Department of Bioinformatics, University of Guelph, Guelph, ON, Canada

**Keywords:** climate change, disease resistance, genomics, machine learning, marker-assisted selection, multi-omics, phenotyping

## Abstract

Anthracnose, caused by *Colletotrichum lindemuthianum*, is a major threat to dry beans (*Phaseolus vulgaris*), causing significant yield losses worldwide. Despite considerable progress in breeding resistant varieties using traditional methods such as phenotypic selection and crossbreeding, the ongoing challenges posed by the pathogen’s genetic diversity and environmental variability call for more sustainable solutions. Traditional breeding methods have made notable advancements, but with the increasing pressure of climate change and evolving disease dynamics, there is a growing need to complement these approaches with modern computational tools. The integration of genomics, phenomics, and bioinformatics has introduced new possibilities in disease resistance breeding. Techniques such as high-throughput sequencing, genome-wide association studies (GWAS), and marker-assisted selection have accelerated the identification of resistance genes, while machine learning and multi-omics approaches provide a deeper understanding of host-pathogen-environment interactions. Therefore, this review aims to provide a comprehensive synthesis of the historical development of anthracnose resistance breeding, highlighting the role of traditional methods and the transition toward computational strategies. It emphasizes how combining both approaches can enhance the development of durable, high-yielding, anthracnose-resistant dry beans, offering more effective solutions to global production challenges.

## Introduction

With the increasing global population intensifying pressure on food systems, there is an urgent need for crops that are affordable, nutritionally dense, and environmentally resilient. This challenge is particularly important considering that nearly three billion people worldwide do not have access to diets that can meet the daily basic nutritional and protein requirements (World Health Organization, 2025). Strengthening food systems therefore require crops that can provide high-quality nutrition while remaining accessible to diverse populations, and adaptable to variable environments.

Dry beans (*Phaseolus vulgaris* L.) play a critical role in addressing this challenge by providing a rich source of plant-based protein and essential micronutrients at relatively low cost (Medendorp et al., 2022; Messina, 2014). They are cultivated across diverse environments worldwide, with major production regions in Asia, the Americas, Africa, Europe, and Oceania (FAOStat, nd). India, Brazil, China, Myanmar, and the United States rank among the leading producers (FAOStat, nd). In many regions, particularly among rural and low-income populations, dry beans serve as a dietary staple and support both smallholder livelihoods and commercial agricultural systems (Beebe et al., 2013).

**Figure 1:**
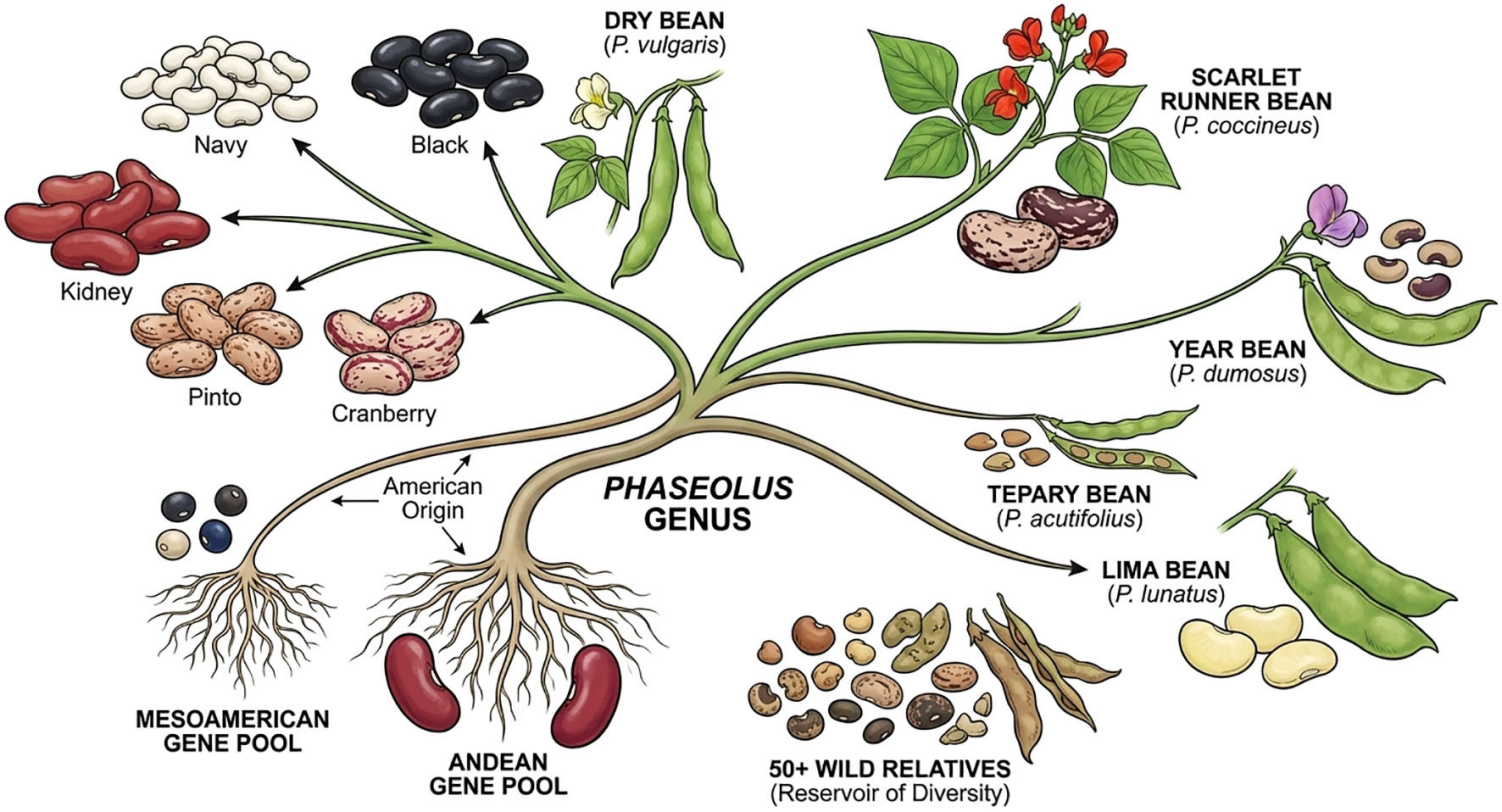
Phylogenetic relationships of cultivated dry beans and their relatives within the *Phaseolus* genus.

Dry beans belong to the genus *Phaseolus*, which includes five cultivated species (**Figure 1**). Among these, dry beans are the most widely cultivated and economically significant species, followed by scarlet runner bean, year bean, tepary bean, and lima bean (Rao, 2020). All cultivated *Phaseolus* species originated in the Americas, with dry beans uniquely domesticated in two major gene pools: the Mesoamerican and Andean (Bellucci et al., 2014; Bitocchi et al., 2013). These two gene pools differ in seed size, growth habit, phenology, and ecological adaptation (Gepts et al., 1986). In addition, more than fifty wild relatives exist (Delgado-Salinas et al., 1999), representing an underexploited reservoir of adaptive genetic diversity (Freytag and Debouck, 2002; Kelly, 2010). Dry beans also show extensive diversity in market classes defined by seed size, color, and end use, including navy, black, kidney, pinto, cranberry, and many regional types, supporting a wide range of culinary and commercial applications (Uebersax et al., 2022).

Over the past decades, breeding and agronomic improvements have contributed to gradual increases in dry bean productivity. However, yield gains have remained modest and highly inconsistent when compared with major cereal crops, as reflected in long-term global production trends (FAOStat, nd). Improvements in yield potential are frequently offset by environmental variability and biotic stress, resulting in substantial yield gaps across regions and seasons (Miklas et al., 2006). Among the major disease constraints affecting dry bean production, pathogens are the most widespread and destructive, particularly under humid and moderate temperature conditions that favor infection (Mahuku et al., 2011; Schwartz et al., 2005; Bitocchi et al., 2013). Major diseases such as anthracnose, angular leaf spot, common bacterial blight, white mold, and bean common mosaic virus occur across diverse regions and repeatedly limit productivity (Mahuku et al., 2011; Schwartz et al., 2005). Among these, anthracnose, caused by *Colletotrichum lindemuthianum* is one of the most destructive and persistent threats to dry bean production. It occurs across tropical, subtropical, and temperate regions and can cause severe defoliation, pod infection, and seed discoloration that can lead to yield losses of up to 100 percent under favorable conditions (Balardin et al., 1997; Padder et al., 2017).

**Figure 2:**
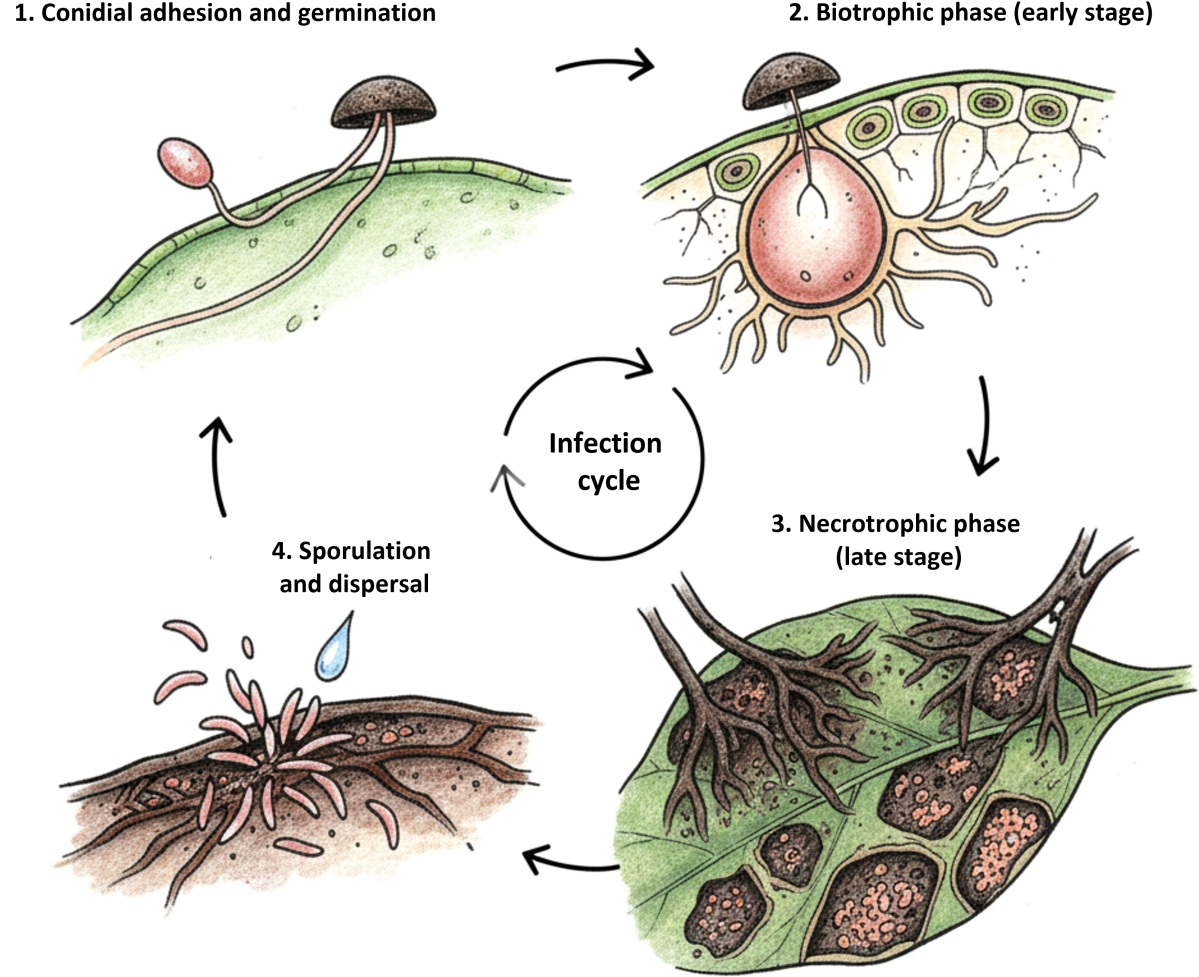
Hemibiotrophic infection cycle of *Colletotrichum lindemuthianum* in dry beans.

Anthracnose is primarily seed borne and shows extensive race diversity, making resistance breeding and disease management particularly challenging. The pathogen is a highly specialized hemibiotrophic fungus that infects dry beans through a two-stage infection process consisting of an initial biotrophic phase followed by a necrotrophic phase (**Figure 2**) (O’Connell et al., 2012; Perfect et al., 1999). After conidia land on host tissues, the fungus forms appressoria that penetrate the epidermis via a penetration peg and establish infection vesicles within host cells (O’Connell et al., 2012; Perfect et al., 1999). This allows the pathogen to evade early defense responses before rapidly switching to destructive necrotrophic growth that causes extensive tissue necrosis and lesion formation (O’Connell et al., 2012; Perfect et al., 1999). More than one hundred physiological races have been reported globally, each defined by differential virulence on specific host resistance genes (Balardin et al., 1997; Padder et al., 2017). This exceptional diversity is driven by mutation, recombination, and strong selection pressure imposed by the widespread deployment of resistant cultivars. In addition, the seed-borne nature of the pathogen enables long-distance dispersal and long-term persistence within production systems, allowing virulent races to spread rapidly across regions (Mahuku and Riascos, 2004).

Owing to its persistent and severe impact on dry bean productivity, breeding programs worldwide have made substantial progress in developing dry bean cultivars with resistance to anthracnose. These efforts have relied largely on the identification and deployment of resistance sources effective against specific pathogen populations (Miklas et al., 2006; Pastor-Corrales et al., 1995). As a result, cultivars with improved resistance have been released across multiple production regions, including high-yielding and resistant varieties such as NUA 48, NUA 64, and RWR 2154 (Kadege et al., 2024), as well as commercially successful cultivars such as Morden003 in Western Canada (Boersma et al., 2013). Field evaluations in Ethiopia have further demonstrated the availability of genotypes with contrasting disease responses and yield performance, supporting the value of resistant germplasm in breeding programs (Hussien et al., 2020). At the molecular level, dominant resistance genes such as *Co-1* in the cultivar Hongyundou (Chen et al., 2017) and marker-assisted backcrossing strategies have enabled the transfer of anthracnose resistance into susceptible backgrounds (Uwera et al., 2021), demonstrating the effectiveness of genetic resistance for improving disease control and maintaining yield performance under disease pressure when properly aligned with local pathogen populations.

Despite these advances in plant breeding, durable anthracnose resistance in dry bean remains difficult to achieve due to the continual emergence and coexistence of diverse pathogen races that frequently overcome race-specific resistance (Del Río et al., 2003; Nunes et al., 2021). In contrast, horizontal resistance is typically characterized by broader but often partial protection against multiple pathogen races. Although it can contribute to more durable resistance in breeding programs, its expression may vary depending on environmental conditions, pathogen diversity, and genetic background (Mulube et al., 2026). In parallel, breeding programs often face challenges in simultaneously improving yield potential, disease resistance, and environmental adaptation, as these traits are influenced by complex genetic interactions and strong genotype × environment effects. This has limited the development of cultivars that combine durable resistance with stable productivity.

In recent years, advances in genomics, phenomics, and data science have led to the emergence of computational breeding approaches. Unlike marker-assisted selection, which focuses on selecting specific markers linked to target genes (Miklas et al., 2006), or conventional genomics-assisted breeding that primarily relies on genomic information to guide selection, computational breeding integrates large-scale genomic, phenotypic, and environmental datasets using advanced statistical modeling and machine learning techniques (Crossa et al., 2017; Varshney et al., 2021; Yoosefzadeh-Najafabadi et al., 2025). These approaches, increasingly adopted in modern breeding programs, enable breeders to better understand complex trait architectures, predict breeding outcomes, and design more efficient breeding strategies for developing resilient cultivars (Crossa et al., 2017; Varshney et al., 2021).

This review aims to provide a comprehensive synthesis of research on anthracnose resistance in dry bean, examining the historical development of resistance breeding, the complexity of resistance-related traits, and emerging global research directions, while highlighting how integrated genomics and phenomics approaches can support the development of cultivars with durable disease resistance and improved yield stability through coordinated, systems-level strategies.

## History of dry beans anthracnose resistance breeding

The development of dry beans genotypes resistant to anthracnose can be a primary objective in legume breeding as it is considered one of the most severe diseases due to its ability to cause significant yield losses (Paulino et al., 2022). To address this, breeders have employed a range of conventional and molecular methodologies to identify, introgress, and stabilize resistance.

Conventional resistance breeding has historically played a central role in anthracnose improvement and continues to evolve. This approach relies on the availability of resistance sources within the primary gene pool, the level of resistance expressed in the cross-compatible germplasm and segregating populations, and the effectiveness of screening under prevailing environmental conditions (Das et al., 2024). The primary gene pool includes *P. vulgaris* itself, encompassing both domesticated landraces and wild relatives. Within *P. vulgaris*, distinct gene pools such as the Mesoamerican and Andean pools contribute substantial genetic diversity, often share common resistance loci, and show no reproductive barriers (Cortinovis et al., 2021; Lobaton et al., 2018). In primary gene pool, researchers have identified some genotypes that serve as important disease resistance donors for anthracnose. For instance, a study on the Andean gene pool reported nine genotypes across diverse market classes with superior resistance to 17 races, demonstrating their value as resistance donors in breeding programs (Mulube et al., 2026). Beyond major resistance genes, a recent study on Andean and Middle American gene pools, novel quantitative resistance loci and defense mechanisms introduced, including age-related resistance and newly identified genomic regions, such as on chromosome Pv10, which can enhance and stabilize resistance when combined with classical *Co* genes (Simons et al., 2022).

Genetic bottlenecks arising from domestication and global dissemination of dry beans have eliminated many alleles associated with stress resilience and disease resistance, resulting in modern cultivars that are genetically narrow compared with their wild relatives (Awale et al., 2024; Cichy et al., 2015). This limitation is especially critical for anthracnose, an exceptionally variable pathogen with hundreds of documented races worldwide, none of which are collectively controlled by resistance genes present in cultivated beans. As a result, resistance derived solely from the primary gene pool has frequently broken down, with widely used genes such as *Co-1* and *Co-2* repeatedly defeated by newly emerging races across multiple continents (Kuwabo et al., 2023; Mwense et al., 2024). These genetic constraints have prompted the use of mutation breeding and interspecific hybridization as complementary strategies to broaden the resistance base in dry beans.

Due to the limited occurrence of spontaneous mutations, chemical and physical mutagens have been used to induce variation and leading to the improvement of diseases resistance in legumes, such as dry beans (Sofkova-Bobcheva et al., 2021), soybean (Zhu et al., 2022), peanut (Han et al., 2017), chickpea (Ahloowalia et al., 2004), pea (Pereira and Leitão, 2010), lentil (Bravo, 1983), and grass pea (Talukdar, 2013). Sofkova-Bobcheva et al. (2021) generated 50 mutant plants using EMS that showed increased tolerance to halo blight (*Pseudomonas savastanoi* pv. *phaseolicola*) and common bacterial blight (*Xanthomonas axonopodis* pv. *phaseoli*) illustrated the potential of mutation breeding to be exploited for improving resistance to anthracnose in common bean (Sofkova-Bobcheva et al., 2021). In soybean, Zhu et al. (2022) reported that a mutant line generated using 150 Gy gamma ray irradiation showed drastic resistance to anthracnose caused by isolate CT5.

Interspecific hybridization and introgression are vital strategies to leverage genetic diversity from wild relatives of dry beans, particularly for developing durable resistance to anthracnose (Awale et al., 2024). Although hybridization with secondary gene pool species is challenging and crosses with tertiary gene pool species often require advanced techniques such as embryo rescue, protoplast fusion, or genetic engineering, these gene pools are strategically important (Lobaton et al., 2018). Species such as *P. coccineus* (scarlet runner bean) and *P. polyanthus* (year bean) from the secondary gene pool and P. *acutifolius* (tepary bean) from the tertiary gene pool harbor high and broad-spectrum levels of resistance absent from *P. vulgaris* (Kumar et al., 2021; Singh and Schwartz, 2010; Mahuku et al., 2002). Moreover, introgression from diverse gene pools enables pyramiding of independent resistance genes from different evolutionary origins, which is widely regarded as the most effective strategy for achieving durable resistance (Costa et al., 2024; Paulino et al., 2022).

When resistance is controlled by a single dominant gene, it can be efficiently transferred through simple backcrossing, whereas resistance governed by multiple genes often requires pyramiding several resistance loci through crosses among different parental lines (Sood et al., 2024). Recurrent selection and backcross breeding are the traditional approaches used for gene pyramiding (Thorat et al., 2024). Recurrent selection is a cyclical and dynamic breeding system designed to gradually increase the frequency of favorable alleles for a specific characteristic within a population (Pádua et al., 2021; Souza et al., 2014). It is primarily recommended for quantitative traits, those governed by a large number of minor-effect genes, but it is also used for traits controlled by numerous major loci (Costa et al., 2024). In dry beans, recurrent selection has been successfully applied to accumulate resistance alleles against highly variable pathogens like anthracnose (Costa et al., 2024), angular leaf spot (Arantes et al., 2010; Librelon et al., 2020; Pádua et al., 2021), and white mold (Leite et al., 2016; Souza et al., 2014). Backcrossing is a fundamental breeding strategy applied to introgress major resistance genes from a donor parent into elite genetic backgrounds and to develop near-isogenic lines (NILs), particularly for anthracnose resistance controlled by high-heritability loci (Costa et al., 2024; Garzón et al., 2008).

The efficiency of recurrent selection and backcross breeding has been further enhanced through marker-assisted recurrent selection (MARS) and marker-assisted backcrossing (MABC). In brief, MARS increases the frequency of multiple favorable alleles with an additive and small individual effects in recurrent crosses (Bernardo and Charcosset, 2006). Although it has not yet been reported in dry beans, some applications in other crops like wheat (Abdulmalik et al., 2017; Rahman et al., 2020), maize (Bankole et al., 2017), Arabica coffee (Saavedra et al., 2023), and cowpea (Boukar et al., 2016) have been reported. In the MABC technique, molecular markers tightly linked to resistance loci enable precise foreground selection of genes such as *Co-4* and *Co-5*, as well as background selection to accelerate recovery of the recurrent parent genome and reduce breeding cycles (Miklas et al., 2006; Miklas et al., 2011). For instance, backcross breeding with the aid of molecular markers was used to obtain advanced pinto bean lines with high levels of common bacterial blight (CBB) resistance with a combination of two quantitative trait loci (QTL), one QTL from the primary gene pool and the other QTL was introgressed from ‘XAN 159’ (Mutlu et al., 2005). This approach has been successfully applied to introgress broad-spectrum resistance alleles from the highly resistant donor cultivar ‘G2333’ into multiple commercial backgrounds, including carioca, black, and navy bean market classes (Miklas et al., 2006; Singh and Schwartz, 2010). Notably, Boersma el. al. (2014) demonstrated the feasibility of pyramiding resistance to anthracnose, CBB, and bean common mosaic virus (BCMV) into elite backgrounds while maintaining yield and seed quality (Boersma et al., 2014). Such multi-disease resistance strategies are particularly important in bean breeding programs where anthracnose often occurs together with other major pathogens. When resistance sources originate from more distantly related or secondary gene pool species, advanced backcross strategies, such as congruity backcrossing, recurrent inbred backcrossing, and advanced backcross QTL analysis, have been employed to overcome interspecific barriers and simultaneously identify favorable alleles from wild germplasm (Beebe et al., 2013; Blair et al., 2018). Despite its effectiveness, backcross breeding is constrained by challenges including linkage drag and the potential disruption of resistance gene clusters during recombination, underscoring the importance of marker-based selection and careful population management (Miklas et al., 2006; Beebe et al., 2013).

For complex traits such as disease resistance, diverse mapping populations are essential for QTL discovery and allele selection to improve cultivars. Bi-parental populations, including recombinant inbred lines (RILs), achieve high mapping resolution through recombination accumulated over multiple selfing generations but remain limited by the genetic diversity of just two parents (Scott et al., 2020; Wang et al., 2011). Nevertheless, RIL populations have been instrumental in identifying anthracnose resistance loci in legumes, particularly through the introgression of novel alleles from landraces and wild relatives. Mulube et al. (2025), characterized Zambian anthracnose isolates and identified resistance QTL in yellow beans, showing that only two of 220 RILs were resistant to all tested races, making these lines valuable resources for cultivar development (Mulube et al., 2025). Kachapulula et al. (2025) evaluated 155 F5:7 RILs and their parents for resistance to seven anthracnose races and identified six QTLs associated with resistance. They suggested that pyramiding two of the major QTLs would provide effective resistance to six anthracnose races in Zambia (Kachapulula et al., 2025). Gonçalves-Vidigal et al. (2020) pyramided resistance to anthracnose (race 73, 2047, and 3481) and angular leaf spot (race 63–39) in 54 RILS of dry beans generated from California Dark Red Kidney × Yolano cross (Gonçalves-Vidigal et al., 2020). RILs are limited by the simultaneous segregation of multiple QTL across the genome, which can mask small-effect loci and complicate the assignment of phenotypic effects to specific genomic regions (Kump et al., 2010; Keurentjes et al., 2007). In contrast, NILs created by backcrossing target regions into recurrent elite parents, provide a clean framework for precise validation and pyramiding (Hawkesford and Griffiths, 2019; Ferreira et al., 2017). Because a NIL typically contains only a single introgression segment from a donor parent, background genetic noise is minimized, significantly increasing the power to detect subtle, small-additive-effect QTL that may be missed in RILs (Kump et al., 2010; Keurentjes et al., 2007). Ferreira et al. (2017) demonstrated the utility of NILs for pyramiding resistance genes in dry beans by developing 16 lines from recurrent parent ‘A25’ through six backcross generations, successfully introgressing and pyramiding stable genomic blocks carrying *Co-2*, *Co-3*, *I*, and *bc-3* alleles conferred anthracnose and viral resistance.

Multi-parent populations, including multi-parent advanced generation inter-cross (MAGIC) and nested association mapping (NAM), are highly effective for pyramiding resistance genes, as they combine multiple founders to generate recombinant inbred lines with broad allelic diversity, enhanced rare-allele detection, and frequent transgressive segregation (Escobar et al., 2022; Odell et al., 2022; Arrones et al., 2020). Some studies have utilized MAGIC populations to improve crops such as dry beans (Ağır, 2023; Escobar et al., 2022; Diaz et al., 2020), soybean (Maranna et al., 2024), and cowpea (Huynh et al., 2018), regarding to diseases tolerance, drought tolerance, and agronomic traits. In dry beans, Escobar et al. (2022), sought to facilitate the mapping and pyramiding of QTLs for white mold resistance in pinto and great northern beans; among 500 lines screened, 19 were highly resistant, with lines WMM-214 and WMM-219 showing consistently high resistance under both greenhouse and field condition. Diaz et al. (2020) developed the first Mesoamerican MAGIC population from eight elite breeding lines, generating 996 RILs via single-seed descent, and identified transgressive segregants, including line MGC583, which showed superior drought yield and enhanced iron and zinc content (Diaz et al., 2020). Ağır (2023) advanced an eight-founder MAGIC population to pyramid white mold resistance QTLs while preserving yield and pod quality, ultimately selecting 15 elite F8 lines. In soybean, Maranna et al. (2024) developed a MAGIC population from eight diverse parents to study (G×E) interactions and charcoal rot resistance; evaluation of 590 F2:8 RILs indicated transgressive segregants, with five lines showing exceptional yield stability, and line ‘G455’ indicating strong disease resistance.

Based on the last recent studies in legumes, NAM populations have been utilized in several landmark investigations to dissect the genetic architecture of complex traits such as agronomic traits in dry bean (Vazin et al., 2018), soil-borne pathogen resistance (Scott et al., 2019; Balk, 2014), yield and biomass in soybean (Balk, 2014; Maranna et al., 2019), phenological traits in lentil (Neupane et al., 2025), late leaf spot resistance, seed and pod weight in peanut (Gangurde et al., 2024, 2020). Soybean NAM populations originate from 40 parents, expressed a higher level of partial resistance to *P. ultimum* var. *ultimum*, while they were highly susceptible to *Py. ultimum* var. *sporangiiferum*, with only six populations there was resistance to more than one pathogen (Scott et al., 2019; Balk, 2014).

## The complex traits which are associated with resistance to anthracnose

**Figure 3:**
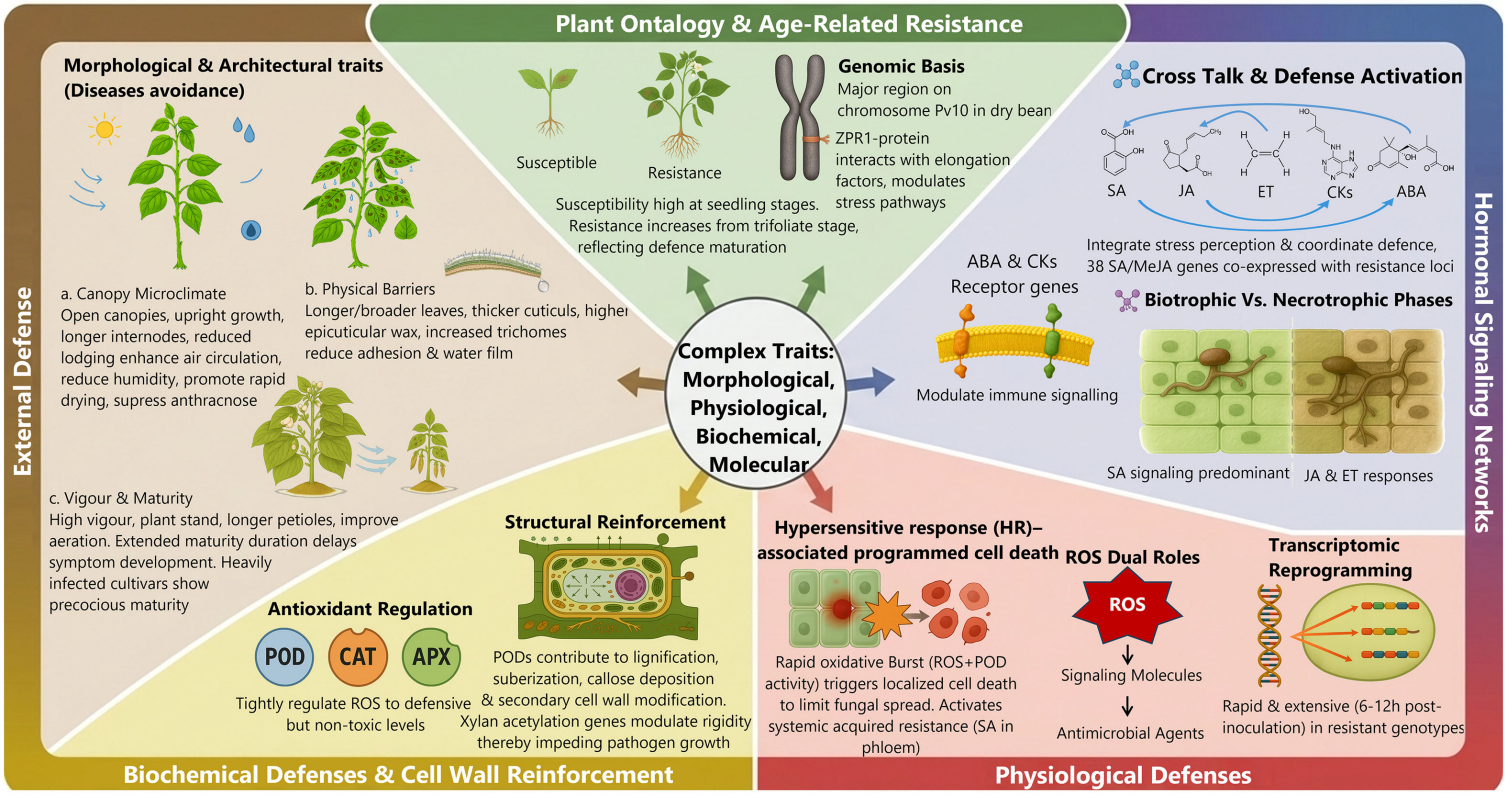
Schematic overview of the complex traits underlying anthracnose resistance in dry beans.

Anthracnose resistance in legumes, especially dry beans, is a typical complex trait controlled by multiple loci and tightly associated with physiological, molecular–biochemical, and morphological mechanisms (**Figure 3**). Resistance expression is strongly influenced by plant ontogeny, with many genotypes displaying high susceptibility at early seedling stages and increased resistance as development progresses, particularly from the trifoliate leaf stage onward (Simons et al., 2022). This phenomenon, commonly described as age-related resistance or adult plant resistance, reflects the maturation of defense capacity and developmental regulation of immune responses (Hu and Yang, 2019). Genome-wide association studies (GWAS) have identified a major genomic region on chromosome Pv10 in dry bean associated with age-related resistance, containing a ZPR1-like zinc-finger domain protein that interacts with elongation growth factors and modulates stress-responsive pathways (Simons et al., 2022). Consistent with this concept, Dhiman et al. (2022) indicated that in dry beans landrace KRC-8 (Baspa), susceptibility at the seedling stage transitions to strong resistance at pre-flowering stages, indicating that developmental resistance can be governed by discrete genetic control, including recessive alleles (Dhiman et al., 2022).

Physiological resistance to pathogens is closely linked to hormonal signaling networks integrating a salicylic acid (SA), jasmonic acid (JA), ethylene (ET), cytokinins (CKs), and abscisic acid (ABA), which crosstalk between these pathways, coordinate stress perception and defense activation (Lovatto et al., 2023; Vaz Bisneta and Gonçalves-Vidigal, 2020; Yang et al., 2019). Chang et al. (2024) identified 38 SA- and methyl jasmonate(MeJA)-responsive candidate genes, which co-expressed with anthracnose resistance loci, highlighting their preferential induction in resistant of dry beans genotypes (Chang et al., 2024). Salicylic acid signaling is predominantly associated with resistance to anthracnose during the biotrophic phase of infection, whereas JA and ET responses become increasingly important as the pathogen shifts toward necrotrophy (Vaz Bisneta and Gonçalves-Vidigal, 2020). Cytokinins signaling has been implicated in legume responses to anthracnose; for example, differential expression of cytokinin receptor genes in partially resistant lentil genotypes, suggests a role in modulating immune signaling, potentially through interactions with SA-mediated defenses (Bawa et al., 2022). During pathogen challenge, resistant bean cultivars such as California Dark Red Kidney showed strong induction of genes involved in hormone perception, notably the ABA receptor gene *Phvul.001G246300* (*PYL5*), highlighting the role of hormone-mediated signaling in resistance modulation (Lovatto et al., 2023).

Beyond foliar defense, resistance also influences the internal movement of anthracnose within the plant. Although the pathogen can colonize stem tissues even in visually asymptomatic plants, the use of healthy seed lots and genetically resistant cultivars remains the primary mechanism for restricting systemic colonization (Halvorson et al., 2021). This suppression of internal colonization can be detected using quantitative polymerase chain reaction (qPCR) as early as 15 days after planting, indicating that resistance operates at both external and internal tissue levels (Halvorson et al., 2021). Among the most effective physiological defenses is hypersensitive response–associated programmed cell death, which is triggered by a rapid oxidative burst involving reactive oxygen species (ROS) and peroxidase (POD) activity (Shafi et al., 2022; Vaz Bisneta and Gonçalves-Vidigal, 2020). These ROS serve dual roles, functioning both as key signaling molecules in defense activation and as direct antimicrobial agents that restrict pathogen growth (Shafi et al., 2023; Hong et al., 2013). Localized cell death limits fungal spread and activates systemic acquired resistance through the SA accumulation in the phloem (Kadege et al., 2024; Spoel and Dong, 2012). Effective resistance further depends on rapid and extensive transcriptomic reprogramming following pathogen challenge. In narrow-leafed lupin infected with anthracnose, resistant genotypes displayed pronounced transcriptomic reprogramming within 6–12 h post-inoculation, whereas susceptible plants showed delayed and weaker responses that were insufficient to arrest infection (Książkiewicz et al., 2022). A notable feature of resistant interactions is the coordinated downregulation of photosynthesis-related genes, which likely redirects metabolic resources toward defense and contributes to the oxidative environment required for hypersensitive cell death (Książkiewicz et al., 2022).

To prevent excessive self-damage, resistant plants tightly regulate antioxidant enzymes, including POD, catalase (CAT), and ascorbate peroxidase (APX), thereby maintaining ROS at defensive but non-toxic level (Shafi et al., 2023). Such regulation of antioxidant enzymes is a hallmark of the biochemical response to anthracnose in dry beans. In the dry beans resistant genotype ‘E10’, early stages of anthracnose infection were characterized by a rapid and robust antioxidant response, particularly a significant increase in APX and POD activities (Shafi et al., 2023). Beyond their role in ROS homeostasis, PODs contribute directly to resistance by reinforcing cell walls through lignification, suberization, and cross-linking of structural compounds, thereby impeding pathogen growth (Almagro et al., 2009). This structural reinforcement is orchestrated at both molecular and biochemical levels, and results in enhanced lignification, callose deposition, and secondary cell wall modification (Książkiewicz et al., 2022; Bacete et al., 2018). These processes are further modulated by genes implicated in xylanacetylation, such as *Phvul.001G246200* in Dark Red Kidney (Lovatto et al., 2023). Enhanced wall rigidity ultimately restricts fungal ingress during the biotrophic stage and contributes to durable systemic resistance (Lovatto et al., 2023; Chakraborty et al., 2019).

Morphological and architectural traits also constitute a critical component of anthracnose resistance in legumes, particularly in dry beans, by acting as primary physical barriers and by modifying the canopy microclimate to limit pathogen establishment and spread (Kadege et al., 2024; LeClair et al., 2015). Because anthracnose requires prolonged surface moisture and high humidity for spore germination and penetration, plant architecture that promotes rapid drying and reduced canopy humidity plays a decisive role in disease avoidance (Escobar et al., 2022). Resistant genotypes often possess longer and broader leaves, thicker cuticles, higher epicuticular wax deposition, and increased trichome density, traits that reduce adhesion and water film formation (Kadege et al., 2024; Gaudencia et al., 2020). Upright growth habits, longer internodes, strong stems, and reduced lodging generate open canopies that enhance air circulation and reduce humidity, and suppress anthracnose development (Kadege et al., 2024). Architecture that facilitates high canopy porosity allows air to circulate effectively through the foliage (Tivoli et al., 2013). This promotes the rapid drying of leaf surfaces after rain or dew, which is essential because the anthracnose pathogen requires moisture for spore germination and transmission (LeClair et al., 2015; Tivoli et al., 2013). In dry beans, determinate and upright indeterminate growth habits (Types I and II) are frequently associated with improved lodging resistance and reduced canopy density (Kelly, 2001), traits that indirectly suppress anthracnose development. Kadege et al. (2024) reported strong positive correlations between resistance to anthracnose and traits such as maturity duration, vigor score, plant stand, internode length, and leaf dimensions in dry beans. Longer petioles and high plant vigor further improve canopy aeration and tolerance to infection stress (Kadege et al., 2024). Extended maturity duration contributes to resistance by delaying symptom development and allowing sufficient time for effective defense activation (Kadege et al., 2024). In contrast, heavily infected cultivars become physiologically weakened and, in an effort to survive and complete their life cycle, accelerate vegetative growth and show precocious reproductive development and early maturity (Kadege et al., 2024). It has also been documented that late maturity can induce resistance to other moisture-favoring diseases in dry beans, such as CBB (Diaz Castro, 2015) and white mold (Escobar et al., 2022; Miklas et al., 2004). Supporting this relationship, Diaz Castro (2015) reported that, late-maturing genotypes in RILs population derived from OAC Rex and OAC Seaforth, showed a greater resistance to CBB than early-maturing lines (Diaz Castro, 2015).

## Anthracnose race determination and the role of differential lines

For the consistent identification and global comparison of the anthracnose races, the need for a standardized classification system was recognized during a CIAT workshop in 1988. This led to the adoption of a uniform differential line system proposed in 1991 by Pastor-Corrales, which has since been widely used for anthracnose race determination worldwide (Pastor-Corrales, 1991) (**Table 1**). The system is based on a fixed set of 12 dry bean differential cultivars having diverse origins and each carrying one or more known resistance genes (**Table 1**). The differential cultivars are always evaluated in the same order and assigned binary values. Following inoculation, each cultivar is scored as resistant or susceptible. The binary values corresponding to susceptible reactions are summed to determine the race designation of the pathogen isolate. For example, if an isolate is susceptible on Michelite, Cornell 49242, and Mexico 222, the corresponding values (1 + 8 + 64) sum to 73, and the isolate is designated as race 73. This standardized nomenclature has enabled consistent communication and comparison of anthracnose races across studies and regions.

**Table 1:** Binary number, gene pool, and resistance genes associated with differential lines/cultivars.

Differential line/cultivar	Binary number (race of anthracnose)	Gene pool	Resistance genes
Michelite	1	Mesoamerican	*Co-11*
Michigan Dark Red Kidney (MDRK)	2	Andean	*Co-1*
Perry Marrow	4	Andean	*Co-1^3^*
Cornell 49242	8	Mesoamerican	*Co-2*
Widusa	16	Andean	*Co-1^5^*
Kaboon	32	Andean	*Co-1^2^*
Mexico 222	64	Mesoamerican	*Co-3*
PI 207262	128	Mesoamerican	*Co-4*
TO	256	Mesoamerican	*Co-4^1^*
TU	512	Mesoamerican	*Co-5*
AB 136	1024	Mesoamerican	*Co-6*
G 2333	2048	Mesoamerican	*Co-4^2^, Co-5. Co-7*

Using this differential system, Padder et al. (2017) reported 182 distinct races worldwide. More recently, Nunes et al. (2021) documented 298 races distributed across 29 countries, highlighting continued race emergence and improved detection efforts. North and South America were identified as major centers of race diversity, together accounting for 222 reported races, likely reflecting both the center of origin of dry beans and long-term host-pathogen co-evolution (Nunes et al., 2021).

The large number of anthracnose races reported worldwide highlights the limitations of relying solely on single race-specific resistance genes in breeding programs. Consequently, breeding strategies increasingly aim to incorporate broader and more durable resistance by combining multiple resistance loci and selecting genotypes that maintain stable resistance across diverse pathogen populations (Miklas et al., 2006; Simons et al., 2022). In practical breeding programs, this often involves evaluating segregating populations against multiple anthracnose races and selecting individuals that consistently express resistance while maintaining acceptable agronomic performance. Because resistance expression can be influenced by environmental conditions and genotype × environment interactions, selection is typically conducted across multiple environments and disease pressures to identify genotypes with stable and durable resistance (Varshney et al., 2021; Kadege et al., 2024).

The distribution of anthracnose races includes both globally prevalent races along with local emerging variants. The most common races of anthracnose that have been reported worldwide are races 73, 65, and 9, which have been documented across North and South America, and Africa (Conner et al., 2020; Zulu, 2011; Gezahegn et al., 2022; Nunes et al., 2021). These races are also frequently referenced in the literature due to their broad geographic occurrence and impact on cultivated germplasm. However, comparatively fewer studies have been conducted on the race diversity in regions of Asia and Africa where dry beans are widely cultivated (Banoo et al., 2020; Bigirimana et al., 2000).

Standardized inoculation and phenotyping procedures using the differential cultivars are typically used for race identification, as described in detail in previous studies (Balardin et al., 1997; Schoonhoven and Pastor-Corrales, 1987). These procedures involve controlled inoculation of seedlings and evaluation of disease symptoms using standardized scoring scales.

## Research trends in anthracnose

A major trend in anthracnose research in dry bean is the shift from reliance on single, race-specific resistance genes toward integrated genetic and phenomics frameworks that better reflect the complexity, durability, and quantitative nature of host resistance (Shafi et al., 2022). Increasing reports of resistance erosion in elite cultivars have prompted a strategic reorientation toward identifying quantitatively inherited resistance components that provide broader, more stable protection across environments and pathogen populations (Kuwabo et al., 2023).

### Genetics and genomics

Dry beans are diploid species with a chromosome number of 22. The genome of the species is approximately 537 Mb; it contains approximately 34,165 genes with about 29,679 protein-coding genes (*Phaseolus Vulgaris*, n.d.). Contained within the genome are disease resistance genes that provide resistance against anthracnose. Many anthracnose resistance genes in dry beans have been uncovered from both Mesoamerican and Andean gene pools; these resistance genes are known as Co genes (Chen et al., 2017). Co genes are often present as gene clusters with tightly linked genes or allelic series and are not often singular genes (Campa et al., 2024). The Co genes can be found throughout the dry bean genome on several chromosomes; they are often found at the ends of chromosomes and are often associated with nucleotide-binding site–leucine-rich repeats (Wu et al., 2017). These genes exhibit dominant patterns of inheritance excluding the only recessive Co gene, namely *Co-8* (González et al., 2015). Co genes are linked to pathogen detection and defense pathways and are often associated with proteins and protein domains which are immune receptors. These resistance genes follow a gene-for-gene model; for every resistance gene in dry beans, there is a counterpart avirulence gene in anthracnose (Burt et al., 2015). Though that is the simplistic explanation of how resistance against anthracnose is observed, it may be much more complicated; certain caveats come into play when considering the alleles of these genes (Burt et al., 2015; Sousa et al., 2015). Co genes are known to have multiple loci and are often part of gene clusters. This in itself adds contradiction to the idea that resistance follows a gene-to-gene model because there is not a specific gene that provides resistance; it is a singular locus with multiple alleles that contribute to anthracnose resistance (Sousa et al., 2015). Another issue arises when considering these multi-allelic loci, that is, the distinction of separate genes which may just be alleles of the same locus (Sousa et al., 2015). Co-9 was thought to be an independent gene, but newer studies have confirmed that it is just another allele of Co-3 (Sousa et al., 2015). Though it is possible that the gene-for-gene model correctly predicts how recognition works (with one resistance gene that may be able to recognize an avirulence gene), resistance to anthracnose in dry beans is much more complex than the idea suggests (Burt et al., 2015). This is the main reason that resistance to anthracnose is treated as a quantitative trait when breeding. It is vital for breeders to breed cultivars with stacked resistance genes that are linked through gene pyramiding to ensure proper precaution against anthracnose infections (Shafi et al., 2024).

Early genomic studies of anthracnose resistance in dry beans during the 1990s and early 2000s laid the foundation for molecular dissection of resistance by introducing DNA marker–based approaches into bean breeding (**Table 2**). During this period, Random Amplified Polymorphic DNA (RAPD) and Restriction Fragment Length Polymorphism (RFLP) markers were extensively used to construct the first genetic linkage maps, identify genomic regions associated with anthracnose resistance, and localize major *Co* resistance genes within the dry bean genome (Adam-Blondon et al., 1994). Although these marker systems were limited by low throughput and reproducibility, they provided critical early insights into the genetic architecture and clustering of anthracnose resistance loci.

**Table 2:** Genomic locations, resistance spectra, and molecular characteristics of major anthracnose (*Co*) resistance genes reported in dry bean (*Phaseolus vulgaris*).

Co gene	Location (Pv chromosome)	Resistance spectra	Associated molecular proteins and structures	Associated mapping and important markers	Molecular mechanisms and functions
*Co-1*	Pv01	Resistance to several races of anthracnose. Alleles provide resistance against specific races.	NB-LRR cluster	Linked to several markers including NDSU_IND_1_50.2219	Race-specific effector sensing and signal transduction
*Co-2*	Pv11	Alleles of the gene provide race-specific resistance.	NB-LRR cluster	Linked to markers SQ4/SCAreoli	Race-specific effector sensing and function in defense pathways
*Co-3*	Pv04	Alleles of the gene provide race-specific resistance.	NB-LRR cluster	Linked to markers 254-G15F_550, SW12	Race specific defense response
*Co-4*	Pv08	Alleles of the gene provide race-specific resistance.	NB-LRR and receptor like kinase cluster	Linked to SCAR markers SAS13, SH18, and SBB14; and other SNPs	Race-specific signaling activation with kinase-associated candidate genes
*Co-5*	Pv07	Alleles of the gene provide race-specific resistance.	Likely NB-LRR cluster	Linked to SCAR maker SAB3; and other QTLs	Part of defense pathways against anthracnose
*Co-6*	Pv07	Alleles of the gene provide race-specific resistance.	Likely NB-LRR cluster	Linked to RAPD makers OPAZ20 and OPZ04_560; and other QTLs	Race specific sensing
*Co-11*	Pv02	Alleles of the gene provide race-specific resistance.	Likely NB-LRR cluster	RAPD marker OPAZ04	Part of defense pathways against anthracnose
*Co-12*	Pv04	Provides resistance against several races.	Not characterized	Not characterized	Part of defense pathways against anthracnose
*Co-13*,	Pv03	Provides resistance against several races.	Not characterized	Linked to marker OV20_680	Part of defense pathways against anthracnose
*Co-14*,	Pv01	Provides resistance against several races.	Not characterized	Same gene cluster as Co-1.	Part of defense pathways against anthracnose
*Co-15*	Pv04	Provides resistance against several races.	Not characterized	Linked to marker g2685_150	Part of defense pathways against anthracnose
*Co-16*	Pv04	Provides resistance against several races.	Not characterized	Linked or marker g2467_800/900	Part of defense pathways against anthracnose
*Co-17*	Pv03	Provides resistance against several races.	Not characterized	Linked to marker B6	Part of defense pathways against anthracnose

Subsequent technological advances in genomics may enabled the development of more robust, sequence-specific markers, greatly improving the precision and efficiency of resistance gene mapping. High-density genotyping platforms, such as the BARCBean6k BeadChip, allowed fine-scale localization of *Co* genes and facilitated the dissection of complex resistance loci (Song et al., 2015). Using BARCBean6k chip, de Lima Castro et al. (2017) discovered the presence of a unique locus in the Paloma cultivar, namely Co-pa, which is linked to the Co-1 cluster and is linked to anthracnose resistance. Along with the discovery of this gene de Lima Castro et al. (2017) found two markers linked to Co-pa, SS82 and SS83, this information is crucial for the development of resistant cultivars as not only do the authors provide a unique resistance gene but they provide a means to identify and track the gene via markers for when breeders would like to breed said gene in to their cultivars. More recently, GWAS have become the dominant approach for discovering anthracnose resistance loci, as single-nucleotide polymorphism (SNP) arrays and genotype-by-sequencing (GBS) enable high-resolution mapping in genetically diverse populations beyond the constraints of biparental crosses (Kuwabo et al., 2023). GWAS analyses across diverse dry bean panels have consistently identified resistance-associated loci on chromosomes Pv01, Pv02, Pv04, Pv05, Pv07, Pv10, and Pv11 (Zuiderveen et al., 2016). Repeatedly detected associations near the Andean *Co-1* locus on Pv01 and within NB-LRR-rich clusters on Pv02 and Pv04 highlight the persistence of major genomic “hotspots” for resistance. In addition, loci on Pv10 associated with age-related and partial resistance emphasize the contribution of developmental regulation to disease response. Together, these findings support breeding strategies that integrate pyramiding of major *Co* genes with minor-effect quantitative trait loci to enhance the durability and breadth of anthracnose resistance (Kuwabo et al., 2023; Simons et al., 2022). More recently, there have even been studies which utilize reference-free k-mer-based GWAS with whole genome sequencing these have provided a more refined resolution as well as structural variation that provides an outlet for pan-genome analysis which is difficult to capture with other techniques (Wiersma et al., 2024). In addition to new technologies for discovery studies, there have been developments in screening technologies as well, one such example is the development of Kompetitive Allele Specific PCR markers that can be used during the marker-assisted selection process for large breeding populations (Zaleski-Cox et al., 2023). The progress from simple markers to highly specific markers and pan-genome approaches has greatly improved the abilities of discovery studies to not only localize markers and characterize anthracnose resistance but also to develop next-generation breeding strategies (Wang et al., 2025; Zaleski-Cox et al., 2023).

Pan-genome approaches are likely to become a more common approach to GWAS discovery studies for anthracnose resistance; not only do they provide researchers with a more complete picture of resistance but they also allow genetic variation to be captured in a new way (Wang et al., 2025). Traditional approaches that utilize reference-based analysis can unintentionally ignore the genetic variation that may be present in dry bean populations, such as land races or different breeding populations (Wang et al., 2025). Pan-genomics utilizes presence-absence variation and structural diversity; this approach may enable the discovery of novel genes. This is particularly relevant to disease resistance research in crops because of the variable nature of disease resistance genes (Wang et al., 2025). Wang et al., 2025, applied this approach to 683 landraces and breeding lines of dry beans and were able to identify over 10000 new genes, including over 300 resistance gene analogs, which have presence-absence variation. These variable genes may be able to unlock novel resources for resistance because they could be absent from the reference but be present in specific landraces or populations of dry beans. The study also demonstrated that the core genome maintains functions that are essential to the plant, but the pan genome contributes significantly to stress responses, which demonstrates the important role that gene presence-absence plays in stress response (Wang et al., 2025). This innovation in genomics research improves the mapping of resistance loci and may be a technique that is adopted in dry breeding programs.

Though there have been significant advances in genomic technologies, the implementation of these technologies in the real world has some real limitations. A significant limitation is accessibility; these technologies are readily available in well-resourced breeding programs, but in regions with poor infrastructure, support, and funding, these technologies are not as accessible (Delannay et al., 2012). This is a significant hurdle for breeding programs in the developing world, as many countries where dry beans play an important nutritional role. Another limitation is a lack of replication; discovery studies which have revealed new anthracnose resistance loci lack environmental replicates (Demirjian et al., 2023). This presents real criticisms of robustness and a lack of data for supporting the use of these new makers in breeding programs around the world. There is also a need to address the differences between controlled experiments and field experiments (Bartoli and Roux, 2017). Field conditions are complex; infection in the field may present differently in comparison to a controlled environment, such as a greenhouse, this may cause the differing observations in regard to anthracnose resistance (Bartoli and Roux, 2017).

The genomic tools discussed have and will aid in the discovery of resistant loci, but these genetic discoveries don’t necessarily have a direct effect on released dry bean varieties. Breeding resistance into varieties is not a straightforward process; when a resistance locus is discovered, it is not directly bred into a variety (Keller et al., 2020; Beaver and Osorno, 2009). Breeding a new variety can take many years in dry beans, and the process is extensive (Beaver and Osorno, 2009). It is very well possible that observed phenotypic resistance was attributed to a variety being resistant to anthracnose, and genomics studies were carried out following the observed resistance to characterize the loci themselves so that they could be made a part of the breeding process by marker-assisted selection (MAS) (Miklas et al., 2006). Nevertheless, there are instances where anthracnose resistance markers have been utilized by breeders to developed resistant cultivars. Boersma et al. (2013) utilized these strategies to successfully breed resistance to anthracnose, common bacterial blight, and bean common mosaic virus into existing cultivars. Boersma et al. (2013) chose to use Morden003, a variety known to be anthracnose resistant, as a parental line and used backcrossing with the resistant varieties as the donor parents and the susceptible varieties as the recipient parents. PCR was then used to test for the presence of molecular markers associated with resistance. Though this is just one example, MAS is a useful tool in breeding processes; though it is not a one-for-all solution, it is still a common part of the whole breeding process and has made the process of breeding resistance into varieties efficient.

As important as it is to study the genetics and genomics of dry beans to address anthracnose resistance, it is just as important to study the pathogen itself due to the co-evolution and gene-to-gene model that resistance against anthracnose follows. According to recent assemblies of anthracnose, the genome of the organism is large and has repetitive and transposable elements (da Silva et al., 2024). The comparison of anthracnose’s genome with other fungal genomes shows that there is a high amount of transport proteins and secondary metabolite clusters within its genome that may be contributing to valerene and its ability to become specialized. This demonstrates genomic plasticity, which may be contributing to its ability for phenotypic diversification (da Silva et al., 2024). These characterizations are in line with the behaviors of the pathogen, where it transitions from a hemibiotrpic pathogen to necrothropic when infecting its host (da Silva et al., 2024). A study by Morelos-Martínez et al., 2024 demonstrated that even within the same country, there were several pathotypes of the fungus which, due to their genetic plasticity, were able to adapt to changes in their host and environment. Furthermore, comparisons between collotrotrum species, including C. lindemuthianum, C. trifolii, C. sidae, C. orbiculare, C. spinosum, C. acutatum, C. gloeosporioides, C. higginsianum, C. graminicola, C. fructicola, C. scovillei, C. sublineola, C. fioriniae, C. truncatum, C. lini, and C. incanum, reveal a pan-genome-like nature with stable housekeeping genes and variable genes within the genomes of all species, and a large amount of CAZyme genes were present in each genome (Morelos-Martínez et al., 2024). The genomes of the species also differ in transposable elements abundance, with CL having the highest TE content. The sizes of the genomes also vary among the species, and the type of CAZymes also differ depending on the lifestyle and host of the pathogens (Morelos-Martínez et al., 2024). These findings demonstrate genomic complexity, pathogen diversity, and the pathogen’s adaptive co-evolution abilities present a need for breeders to adopt strategies that anticipate the ongoing host-pathogen co-evolution.

Although there is information known about anthracnose resistance in dry beans, there is quite a bit that is unknown. One of the main knowledge gaps is the molecular mechanisms associated with Co genes (Chang et al., 2024). Though many Co genes are known to be part of defense pathways, their exact role in the pathways has not been established (Chang et al., 2024). This indicates that there is still research required to address the connections between gene expression and function. A study by (Simons et al., 2022) demonstrated that disease resistance against anthracnose can differ in dry beans depending on growth stages. There is a gap in knowledge on how differences in growth stages, race, and even environment can affect resistance, which, if addressed, would provide a more robust picture of anthracnose resistance in dry beans. Another limitation, as mentioned previously, is the evolution of anthracnose pathotypes and the co-evolution of the fungus alongside its host; this knowledge gap represents the lack of a link between pathogen diversity and the resistance of the host (Simons et al., 2022). These are just some limitations that arise in genomic studies pertaining to anthracnose resistance in dry beans, and addressing these knowledge gaps may be essential to advancing anthracnose resistance in dry bean cultivars.

### Phenomics

In modern agricultural systems, traditional disease management strategies such as crop rotation, fungicide application, and the deployment of single resistance genes have contributed significantly to disease control but are increasingly challenged by the rapid adaptation and diversification of pathogen populations. Anthracnose in dry beans exemplifies this complexity, as genetically diverse pathogen races can erode the effectiveness of previously successful resistance sources over time. This ongoing host–pathogen co-evolution highlights the need for breeding strategies that move beyond static resistance deployment and instead incorporate continuous, quantitative, and biologically meaningful measurements of plant responses to infection. Importantly, these measurements differ fundamentally from traditional visual or categorical disease ratings by capturing temporal dynamics, physiological responses, and trait interactions that more accurately reflect resistance expression under diverse environments.

Phenomics addresses this challenge by shifting plant breeding from qualitative observation toward data-driven, systems-level measurement of how plants grow, function, and respond to stress across environments. Rather than focusing on a few traits scored at isolated time points, phenomics captures the full expression of the genome as it unfolds in real time under fluctuating environmental conditions. This concept of the phenome, defined as the total set of observable characteristics produced by the interaction between genotype and environment, provides a biological counterpart to genomic information, which alone cannot predict how a plant will perform in the field (Soulé, 1967). When phenomics is integrated with molecular data, breeders can link specific genetic variants to measurable physiological and morphological outcomes, enabling a more precise understanding of why certain genotypes succeed or fail under disease pressure.

**Figure 4:**
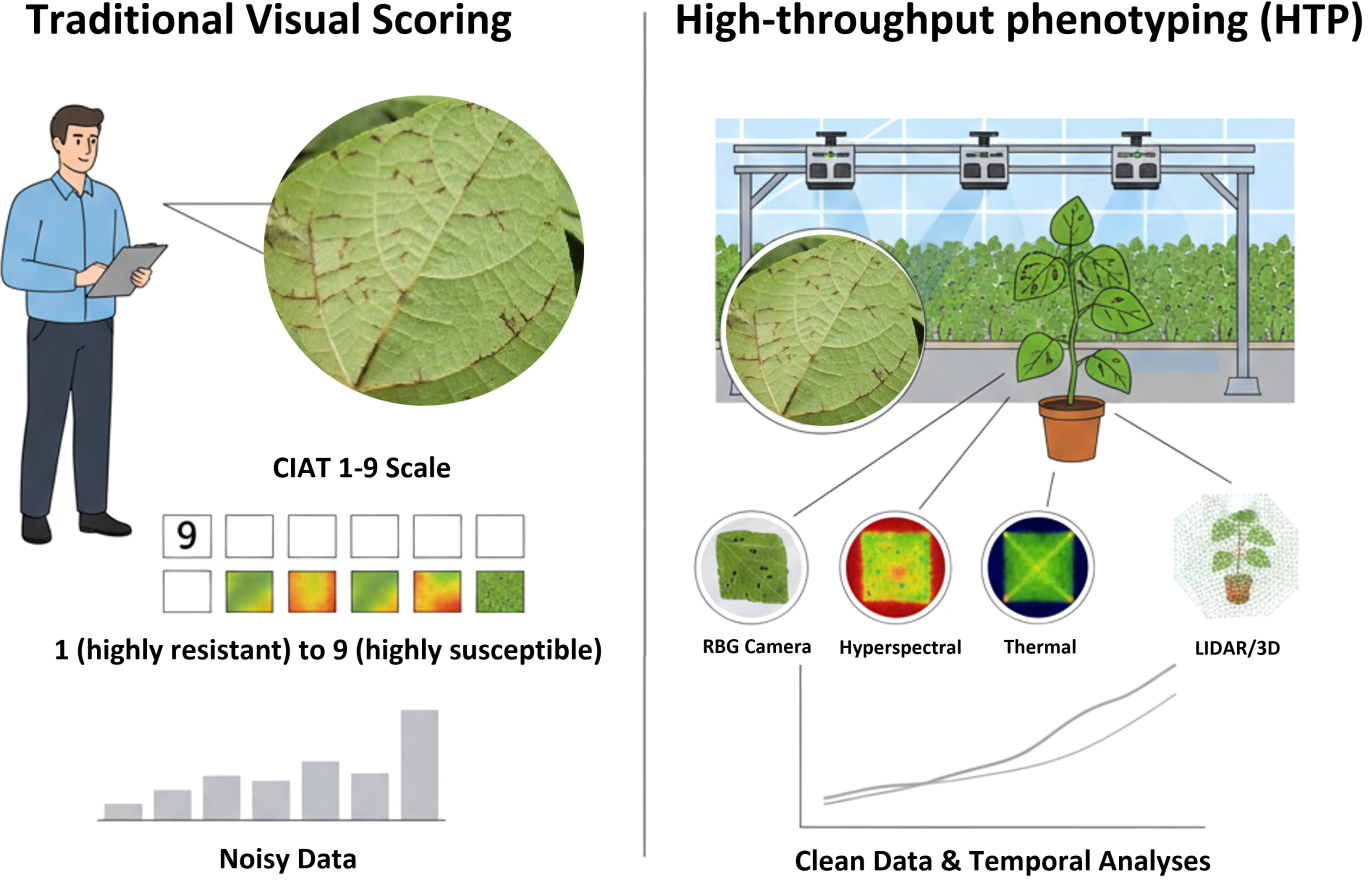
Comparison of traditional visual scoring and high-throughput phenotyping approaches for assessing anthracnose resistance in dry beans.

Traditional disease phenotyping methods cannot capture this complexity. Visual disease scoring relies on human judgment to estimate symptom severity, typically based on lesion coverage, tissue discoloration, or plant death (Mahlein, 2016). In dry beans anthracnose screening, the CIAT 1 to 9 (**Figure 4**) scale translates these observations into numerical values based on the proportion of affected leaf veins and stem tissues (Degu et al., 2024). While these scales provide standardization, they do not measure the underlying biological processes driving symptom development. Moreover, visual assessments are sensitive to rater bias, lighting conditions, canopy density, and scoring time, which introduces substantial noise into phenotypic datasets (Bock et al., 2008, Bock et al., 2010; Newton and Hackett, 1994; Nutter et al., 1993; Steddom et al., 2005). This noise directly limits the power of genetic mapping and genomic selection because inaccurate phenotypes weaken the association between genotype and trait. Conventional plant disease assessments rely on visual estimates of symptoms (lesions, blight, galls, tumors, cankers, wilts, rots) and other signs of the presence of pathogens (e.g., spores, mycelium) by trained experts following standardized rating guidelines (Mahlein, 2016).

Modern high-throughput phenotyping (HTP) systems have made this integration possible by generating continuous, objective measurements of plant traits at unprecedented spatial and temporal resolution (Zhao et al., 2019). These systems combine visible spectrum imaging with hyperspectral, multispectral, thermal, and fluorescence sensors to quantify leaf reflectance, tissue temperature, chlorophyll activity, canopy structure, and growth dynamics (Zhao et al., 2019). Three-dimensional reconstruction and LiDAR further allow the assessment of biomass accumulation, canopy architecture, and plant height without physical contact. Because these measurements are collected repeatedly throughout the growing season, they not only determine whether a plant is diseased but also how rapidly infection progresses, how strongly it disrupts physiological processes, and how effectively the plant compensates through growth and resource allocation. Despite the clear advantages of high-throughput phenotyping (HTP), there can be limitations such as cost, reproducibility, and noise in disease scoring. The high cost of HTP systems often limits their adoption in plant breeding programs, as conventional field phenotyping is among the top three most costly activities of breeding efforts (Hoyos-Villegas et al., 2025). In developing countries, adopting new phenotyping technologies can be challenging due to prolonged financial constraints, a lack of skilled workers, and infrastructure and sourcing limitations, all of which may limit their affordability and adoption (Hoyos-Villegas et al., 2025). Evidence from Nigeria shows that 40% of universities and National Agricultural Research Systems (NARS) were unsuitable for modern agricultural research due to unreliable electricity, limited communication systems, and shortages of chemicals and consumables (Adisa and Toro, 2016). Challenges in reproducibility can result from a lack of standardization and compatibility among phenotyping platforms and data formats, which can complicate data sharing and integration across breeding programs and research groups (Liu and Xu, 2023; Medic et al., 2023). With increasing UAV weight, users may also be required to obtain a drone pilot certificate, which can add additional training requirements and costs (Colomina and Molina, 2014). UAVs without gimbal stabilization can lead to image blur and distortion, reducing the quality and reliability of orthomosaics used for disease severity assessment (Hoyos-Villegas et al., 2025). This temporal dimension is particularly important for disease resistance, which often involves delaying symptom development, restricting lesion expansion, or maintaining photosynthesis despite infection rather than simply preventing infection altogether.

Imaging-based phenomics overcomes these constraints by detecting disease-related changes that are invisible or ambiguous to the human eye. Hyperspectral sensors can identify subtle shifts in pigment composition, water content, and cellular structure that occur when pathogens disrupt leaf metabolism (Junges et al., 2020; Mahlein et al., 2019). Thermal imaging demonstrates altered transpiration caused by stomatal closure or vascular blockage, while chlorophyll fluorescence exposes declines in photosynthetic efficiency that precede visible necrosis. Together, these signals provide a multidimensional fingerprint of plant health that allows precise and uniform quantification of disease severity, infection timing, and host response. Instead of scoring symptoms after damage has occurred, breeders can use these technologies to measure how effectively a genotype resists or tolerates infection during the earliest stages of pathogen invasion.

High-throughput phenotyping systems provide automated, objective detection systems for early identification and quantification of plant diseases (Mahlein, 2016). Since these imaging techniques generate terabytes of imaging data daily, this cannot be processed manually, resulting in the use of predictive artificial intelligence (AI) algorithms such as machine learning for phenotype identification and severity estimation (Gill et al., 2022; Najafabadi and Jackson, 2025). AI has been rapidly integrated into plant phenomics to accelerate data analysis, automate sensing, and support decision-making in genomic selection and phenomic prediction (Wang et al., 2026). Although limited sample sizes and complex trait interactions can still constrain model performance (Mansoor et al., 2025), the combination of large phenotyping datasets with advanced AI continues to improve prediction accuracy and breeding efficiency.

In dry bean breeding, this capability is especially important for anthracnose, a disease whose severity is strongly influenced by both genotype and environment. Resistance does not arise from a single protective mechanism but from coordinated biochemical, structural, and physiological responses that limit pathogen growth and preserve plant function. Biochemical traits such as phenolic compounds, phytoalexins, and defense-related proteins determine how quickly a plant recognizes a pathogen and activates immune pathways (Robles-Zazueta et al., 2022). Morphological features including leaf size, canopy density, stem architecture, and pod orientation shape the microclimate within the canopy and influence how spores disperse and infect new tissue (Gaudencia et al., 2020). Physiological traits such as photosynthetic capacity, nitrogen use efficiency, and stomatal regulation determine whether a plant can sustain growth while mounting a defense response (Sudhakar et al., 2016). Together, these trait layers explain why some genotypes maintain yield under disease pressure while others collapse even when symptom scores appear similar. Leaf morphology provides a clear example of how phenomics links structure to disease response. High resolution imaging allows these traits to be measured precisely and repeatedly, enabling the calculation of lesion growth rates, infected leaf area, and canopy-level disease burden. When these structural measurements are integrated with hyperspectral or fluorescence data, researchers can directly relate physical damage to physiological impairment, revealing which genotypes sustain photosynthesis despite infection and which rapidly lose functional leaf area.

Leaf width and length are key morphological traits in beans, as anthracnose appears as dark, linear lesions ranging from black to brick-red on the lower leaf surface and along the veins at the trifoliate and primary leaf stages, making these traits useful indicators of genotype resistance against anthracnose (Mahlein, 2016; Sharma et al., 2007). Imaging technologies such as hyperspectral and chlorophyll fluorescence sensing can detect physiological disruptions caused by infection, including changes in photosynthetic efficiency, pigment composition, and water status before visible symptoms appear (Mahlein et al., 2019). Rather than solely relying on visual symptom expression, hyperspectral imaging analyzes reflectance across specific spectral bands to capture pathogen-induced alterations in leaf tissue structure and biochemical composition during the earliest stages of disease development (Junges et al., 2020). Together, these approaches allow researchers to distinguish between genotypes that exhibit delayed symptom expression, sustained physiological function, or early metabolic disruption, providing a more mechanistic understanding of disease resistance beyond visible lesion severity.

Anthracnose infection disrupts multiple layers of plant function, making resistance a complex trait that cannot be fully captured by single visual disease scores. Consequently, phenomics-based analyses emphasize biochemical, morphological, and physiological traits that collectively describe how plants respond to infection over time. Biochemical traits, including defense-related metabolites, phytohormones, and antimicrobial compounds, reflect the speed and intensity of pathogen recognition and immune signaling activation that constrain fungal growth (Robles-Zazueta et al., 2022). Morphological traits such as biomass accumulation, plant height, leaf area, canopy architecture, and pod structure influence canopy microclimate and spore dispersal, thereby shaping disease progression within plant stands. Kadege et al. (2024) demonstrated that anthracnose-resistant dry bean lines can be more effectively differentiated using combinations of phenotypic traits rather than relying solely on visual disease ratings. By jointly evaluating plant vigor, maturity, yield-related traits, and disease severity, the study identified genotypes that maintained growth and productivity under infection pressure, allowing breeders to distinguish true physiological resistance from symptom avoidance or escape mechanisms. This multi-trait phenotypic framework provides a more robust and biologically meaningful basis for selection and cultivar advancement in dry bean breeding programs.

When phenomics is integrated with genomics, transcriptomics, proteomics, and metabolomics, it forms a unified systems-level framework for understanding how genetic variation translates into disease resistance and yield stability under realistic field conditions (Ngomle et al., 2025). In this context, phenomics serves as the critical bridge between molecular variation and agronomic performance, enabling the validation of candidate genes and pathways in production environments. Together, multi-omics approaches have transformed plant pathology and resistance breeding by improving trait resolution, accelerating selection, and supporting early disease detection (Ngomle et al., 2025). Nevertheless, important challenges remain, including the difficulty of accurately modeling complex trait interactions with machine learning approaches and the labor-intensive nature of phenotypic data collection, which can limit dataset size and environmental coverage (Mansoor et al., 2025; Yoosefzadeh Najafabadi and Torkamaneh, 2025). Despite these limitations, phenomics remains a cornerstone of modern resistance breeding, as advances in high-throughput imaging, AI, and integrated multi-omics continue to improve the efficiency, accuracy, and scalability of developing durable anthracnose-resistant dry bean cultivars with stable productivity.

### Multi-omics

In recent years, research trends have been gravitating towards a multi-omics approach where integration of systems biology is being used to address anthracnose resistance in dry beans. A multi-omics approach combines multiple “omics” fields such as genomics, transcriptomics, metabolomics, proteomics, phenomics, and enviromics to provide a comprehensive understanding of genetic variation and its effect on phenotypes when challenged with disease (Cortés et al., 2023; Mahmood et al., 2022). Genomics provides the foundation of resistance as it gives insight into the genetic components that are involved in anthracnose resistance (Kuwabo et al., 2023). Through techniques such as GWAS and linkage mapping, several resistance loci, Co genes, have already been identified, and new resistance loci continue to be uncovered. The field of genomics plays the role of identification of genes and their localization, as well as possible protein and pathway associations in this multi-omics approach.

Transcriptomics, another component of the multi-omics approach, provides a dynamic view of disease response. The field explores gene expression in response to infection at various times with various genotypes and is a critical step to bridging the gap between pathways of infection response and how resistance is reflected in gene expression and genetic variation (Chang et al., 2024). Techniques such as RNA-seq are commonly applied in transcriptomics as they provide a robust view of differential gene expression and even co-expressed genes with known resistance genes (Chang et al., 2024). Network-based analyses, particularly weighted gene co-expression network analysis (WGCNA), have enabled the identification of resistance-associated gene modules enriched for protein kinases, transcription factors, hormone signaling components, and classical resistance genes (Niu et al., 2024; Wu et al., 2022). Therefore, transcriptomics is a crucial part of the multi-omics approach because of the abundance of knowledge it can provide about molecular responses to infection.

Proteomics can be used to further validate findings from transcriptomics by confirming the presence of proteins related to resistance. Though the research in proteomics related to anthracnose in dry beans is limited, there have been similar studies on related plant-pathogen systems, which demonstrate the importance of differential protein accumulation and its role in providing effective resistance against pathogens (Pandey et al., 2023). Proteomic profiling studies in dry beans could provide important information about plant responses to anthracnose infections; they would provide validation and allow the refinement of candidate resistance genes from previous genomics and transcriptomics studies (Pandey et al., 2023).

Metabolomics provides data regarding the biochemical response that dry beans have to an anthracnose infection. As with proteomics, research that explores the metabolic profile of dry beans in response to an anthracnose infection is limited. Though this is the case, a study by Shafi et al., 2024 highlights the importance of metabolic studies as they provide an outlet for identifying biochemical responses to infection. In the study Shafi et al., 2024 were able to mark the changes in antioxidant levels in susceptible and resistant plants, and changes in enzymatic activity. The study demonstrated that there is a large increase in number of antioxidants and enzymatic activity (in the enzymes superoxide dismutase, ascorbate peroxidase, and glutathione reductase) of in resistant genotypes. Such studies can aid in the identification of valuable biochemical markers that are part of resistance pathways.

Phenomics acts as a quantitative link between molecular signals and phenotypic disease responses. Through high-throughput phenotyping of infected breeding lines, a significant variation in disease severity and correlated quantitative traits such as maturity, plant architecture, and vigor can be measured, which demonstrates the variation of disease responses of several different genotypes of dry beans (Kadege et al., 2024). As described previously in the genomics section of this review, there are many applications for phenomics in disease resistance breeding in dry beans (Junges et al., 2020; Kadege et al., 2024; Mahlein et al., 2019). Phenomics acts as a means of objective trait scoring, which can be associated with predictors of disease response and resistance.

Enviromics can add a layer to the previously mentioned “-omics” that provides valuable G×E interaction data. Environics can provide a means to measure the performance of resistant genotypes when faced with several unique weather patterns, soil properties, and other environmental effects (Resende et al., 2021). Resende et al., 2021 suggest that Environics can be applied to manage biotic stressors such as plant diseases like anthracnose because it can provide breeders with information on the characteristics that resistant genotypes may display in field environments. This, in combination with genotypic and phenotypic data, can be integrated into breeding pipelines to allow breeders to predict the performance of genotypes with higher accuracy (Resende et al., 2021). Enviromics, in turn, allows breeders to predict how environmental factors can influence anthracnose resistance in dry bean populations.

Additionally, when considering breeding for disease resistance genotype, it is important to consider genotype ´ environment ´ pathogen interactions as well as G´E interactions (Balotf et al., 2025). The combination of multiple omic fields can be applied to capture these interactions. Genomics can provide genetic loci related to infection and resistance, transcripomics can capture variation in gene expression in infected plants, proteomics and metabolomics, and work in conjunction to characterize the biochemical pathways active when a dry bean crop is fighting an anthracnose infection, phenomics would provide a means to capture the phenotype of diseased plants across multiple environments, and enviromics can capture the environmental covariates (Ngomle et al., 2025; Balotf et al., 2025; Resende et al., 2021). The information that is produced via these omics can then be integrated into prediction models, which could provide breeders with a comprehensive picture of how a given cultivar may react to infection without having to carry out a field experiment.

Although the omic fields discussed in this paper provide an understanding of the possible uses of the omics in a breeding context, the use and coverage of these omics’ technologies in current breeding programs are uneven. Genomics is currently the most commonly applied and utilized omics in breeding programs; MAS are ubiquitous in not only anthracnose-related dry bean breeding but also breeding as a field itself (Wang et al., 2023). Transcriptomics, proteomics, and metabolomics can provide valuable insight into the biochemical workings of a crop’s functions, but currently these fields are not focused on in breeding programs due to their limitations (P. Wang et al., 2024; Panahi et al., 2025; Wang et al., 2023; Resende et al., 2024; Komatsu et al., 2013; Pieruschka and Schurr, 2019; Hao et al., 2025). Transcriptomics is limited by data scarcity and the complex intricacies of connecting phenotypic traits to the genetic mechanisms that affect the trait (Panahi et al., 2025). Proteomics is limited by dependency on genomic data for annotation and the lack of technologies and information on how unique post-translational modifications can affect the activity of proteins (Komatsu et al., 2013). Metabolomics is heavily limited by sample collections because of the volatile nature of metabolites and the difficulties of analyzing the sample and the dynamic nature of metabolomics through statistical methods (Hao et al., 2025). Phenomics has its own challenges, such as spatial limitations when wanting to do a comprehensive phenotypic study, the number of genotypes one can use is limited by the space one has to grow them, and the challenge of tracking the plastic nature of plants (Pieruschka and Schurr, 2019). One of the main limitations of enviromics is the amount of data; enviromics data is large-scale data and requires advanced computational infrastructure for analysis (Resende et al., 2024). These are just some of the limitations of the omics fields discussed in this paper; because of these challenges, most of the omics are severely underutilized in the crop breeding process.

Integration of data from these various fields through association by network analyses and machine learning algorithms allows for the development of system biology models that can connect genomic variants to expression patterns, protein functions and molecular pathways, and phenotypic responses of various genotypes (Yoosefzadeh-Najafabadi, 2025b). The merging of these fields to form a multi-omics approach can, in turn, improve marker-assisted selection and genomic prediction models for anthracnose resistance in dry beans.

## Future perspectives and challenges

### Advances in genomics and biotechnology

As anthracnose continues to evolve and overcome existing host resistance mechanisms, it is crucial to continue progressing in breeding strategies that can respond to pathogen variability and evolution, ensuring long-term resistance in cultivars (Benchimol-Reis et al., 2025). Emerging molecular marker technologies and high-throughput sequencing platforms are accelerating dry bean improvement and enhancing resistance to fungal diseases (Benchimol-Reis et al., 2025). In particular, the integration of pan-genomes and high-quality reference sequences is expected to enable the identification of functional resistant alleles and to confirm candidate genes associated with anthracnose resistance (Benchimol-Reis et al., 2025). In addition, gene-editing technologies such as CRISPR-Cas9 provide promising opportunities for precise manipulation of resistance loci and rapid trait improvement in dry bean breeding programs.

### Breeding strategies for durable resistance

Future progress in anthracnose resistance breeding will depend on a deeper understanding of the genetic architecture and inheritance patterns of resistance loci. The development of molecular markers linked to anthracnose resistance genes with different modes of action may facilitate gene pyramiding strategies aimed at improving durability of resistance (Simons et al., 2022). In addition, integrating desirable agronomic traits such as yield potential, seed quality, and plant architecture with disease resistance will remain a priority in breeding programs. Previous studies have demonstrated that anthracnose-resistant cultivars can also exhibit favorable agronomic traits that contribute to improved productivity when incorporated into breeding pipelines (Boersma et al., 2014).

An important factor which breeders will need to consider as time goes on is climate change. Climate change can cause an increase in the variability of traits and plant reactions to an anthracnose infection (Garrett et al., 2006). Environmental stress may change how a plant defends itself against an anthracnose infection, this may mean a change in mechanisms such as the production of phenols, changes in enzyme activity, and possibly changes in plant morphology (Son and Park, 2022). When the environment is variable, stability can be provided by genetic components, because this is the case there will be a need for continued genetic research (Pour-Aboughadareh et al., 2022).

### Role of phenomics and integrated data approaches

Advances in high-throughput phenotyping technologies are expected to play an increasingly important role in understanding the complex traits associated with anthracnose resistance. Recent studies suggest that resistance is linked to networks of traits such as plant vigor, canopy structure, maturity, and yield stability that interact with environmental conditions (Kadege et al., 2024). Moving beyond traditional visual disease scoring, modern phenomics approaches now incorporate high-throughput imaging, sensor-based measurements, and computational analyses to capture temporal disease progression and physiological responses at multiple biological scales (Hesami and Yoosefzadeh-Najafabadi, 2025). These approaches allow researchers to analyze complex phenotypic variation and better understand the genetic mechanisms underlying disease resistance.

The integration of phenomics datasets with genomic sequencing, quantitative trait mapping, and genomic selection pipelines offers a powerful framework for improving prediction accuracy and accelerating the deployment of resistance alleles for disease resistance and yield stability (Varshney et al., 2021). Furthermore, phenomics-driven evaluation of breeding methodologies can help optimize or replace labor-intensive strategies such as repeated backcrossing and manual selection by providing more comprehensive assessments across multiple traits and environments (Ahmar et al., 2020; Yoosefzadeh-Najafabadi, 2025a). Ultimately, integrating phenomics with advances in genomics, molecular breeding, and improved disease management strategies will be essential for developing dry bean cultivars with durable anthracnose resistance and improved resilience under evolving pathogen and climate pressures.

## Conclusion

Breeding programs targeting anthracnose resistance in dry bean increasingly integrate traditional and computational tools to achieve durable, high-yielding cultivars that can keep pace with rapid pathogen evolution and environmental change. Conventional methods such as recurrent selection, backcrossing, gene pyramiding, and the strategic use of primary, secondary, and tertiary gene pools remain central to cultivar development, providing the genetic foundation for resistance deployment. At the same time, genomics-assisted approaches, including QTL mapping, GWAS, and marker-assisted selection, are routinely used in many programs to identify and track resistance loci, enabling more precise introgression and pyramiding of Co genes and quantitative resistance alleles. Emerging phenomics platforms and high-throughput disease phenotyping offer the potential to quantify resistance expression dynamically and reduce subjectivity in disease scoring, although their adoption is currently constrained by cost, infrastructure, and expertise in many regions. Looking ahead, the integration of multi-omics datasets with advanced statistical modelling and artificial intelligence is expected to further enhance prediction accuracy and selection efficiency, gradually transitioning breeding pipelines from empirical selection toward data-driven, computational breeding frameworks that better anticipate host–pathogen co-evolution and support more resilient dry bean production systems worldwide.
